# ASO Author Reflections: Hormonal Status May Impact Outcomes in Peritoneal Mesothelioma After CRS/HIPEC

**DOI:** 10.1245/s10434-026-19503-6

**Published:** 2026-03-25

**Authors:** Kaelyn C. Cummins, Samantha M. Ruff

**Affiliations:** Department of Surgery, University of Virginia Health, Charlottesville, USA

## Past

Epithelioid peritoneal mesothelioma is a rare malignancy for which cytoreductive surgery and hyperthermic intraperitoneal chemotherapy (CRS/HIPEC) remains the standard of care in appropriately selected patients.^1^ Male sex has consistently been associated with worse survival;^1–3^ however, the biological mechanisms underlying this disparity remain poorly defined. Sex hormones have been shown to influence tumor development, progression, and therapeutic response in several malignancies, including breast, prostate, and colorectal cancers, indicating that hormonal signaling pathways may contribute meaningfully to oncologic behavior.^4^ Prior small studies have suggested a potential role for sex hormones in the disease biology and outcomes of peritoneal mesothelioma,^2^^,^^3^ yet these observations have not been evaluated in a large, contemporary national cohort.

## Present

Using the National Cancer Database, we evaluated 1868 adult patients with epithelioid peritoneal mesothelioma who underwent CRS/HIPEC.^5^ Even among patients with favorable clinical profiles, CRS/HIPEC appeared underutilized. Male sex was independently associated both with receiving CRS/HIPEC and worse overall survival. To further explore the potential influence of hormonal status, we constructed a sex-age composite variable using age thresholds of ≤ 40 and ≥ 60 years as proxies for pre- and postmenopausal states. Premenopausal women demonstrated significantly improved overall survival compared with postmenopausal women, younger men, and older men, suggesting a potential prognostic role for sex hormones in this patient population.

## Future

Further investigation is needed to elucidate the biological mechanisms underlying these observed sex- and age-based disparities. Prospective studies incorporating direct hormone measurements and characterization of hormone receptor expression and downstream signaling pathways are warranted. In parallel, efforts should focus on understanding practice patterns contributing to sex-based disparities in CRS/HIPEC utilization, particularly potential undertreatment among appropriate candidates. Clarifying both biological and systems-level drivers of disparity will be essential to optimizing patient selection and ensure equitable delivery of CRS/HIPEC.
